# Editorial: Genetics and justice: the implications of large-scale genome sequencing initiatives in the global south

**DOI:** 10.3389/fgene.2025.1703507

**Published:** 2025-10-08

**Authors:** Michel Satya Naslavsky, Tayyaba Jiwani, Ernesto Schwartz-Marin

**Affiliations:** ^1^ Human Genome Research Center, Biosciences Institute, University of São Paulo, São Paulo, Brazil; ^2^ Hospital Israelita Albert Einstein, São Paulo, Brazil; ^3^ University of Exeter, Exeter, United Kingdom

**Keywords:** population genomics, ancestry and race, justice & Inequalities, global south, bioethics

This Research Topic presents interdisciplinary interrogations of population genomics programs, as embedded in local and global hierarchies of knowledge, power, and capital. It also evaluates different approaches taken to address their criticisms, in attempts to develop a more ethical and just genomics.

Populations across the global South are increasingly being recruited into national and international genomics programs with promises of a healthier and more prosperous future. The lack of population diversity is considered a limitation of existing genetic datasets. Increasing population diversity, in terms of genetic ancestries and lifestyles, is expected to yield new insights into human origins and complex genetic associations with disease. Such analyses are thus deemed desirable from scientific, medical, and economic perspectives.

In this Research Topic, de Oliveira et al. argue along these lines for a greater representation of Latin American and Caribbean (LAC) populations in global genomics studies. Gooden and Thaldar make a similar case for the inclusion of “persons of African origin”, proposing the creation of a “large-scale, open-access genomics database of South Africans”. Acknowledging concerns around ethics, privacy and informed consent, their paper proposes a legal and policy solution in the form of “open consent” with deep community engagement.

Simultaneously, attempts to increase diversity in global genomics have been strongly criticised for reproducing and reifying colonially ordered racial classifications. Despite geneticists broadly dismissing the biological reality of race, critical scholars have pointed out that contemporary studies nevertheless view human diversity through the lens of continental racial stocks. In Dauda et al., a group of leading geneticists, ethicists, and sociologists examine the use of the alternative term “ancestry” in genetic studies “as a more objective and less objectionable population descriptor than race or ethnicity”. Their analysis demonstrates that despite attempts to distance ancestry from race/ethnic categories, there are “many instances of slippage”, which highlight the need for greater “conceptual clarity”.

Similarly, King et al. call for opening a dialogue “about how and why race is employed” in environmental epigenetics. Though the field offers valuable opportunities to investigate the links between environmental stressors and health disparities, they argue that uncritically employing race as a covariable, without an understanding of the role of structural racism in health, risks perpetuating “harmful biases”. They call for greater interdisciplinary exchange between epigeneticists and social scientists to enrich studies on health outcomes with “more detailed social metrics”.

It is also worth a reminder that the shadow of colonialism and race science on genomics cannot be overcome through the use of better terms alone. Instead, as critical scholars have shown, there is a need for genetic science to concretely understand and challenge the colonial histories, politics and epistemologies that structure the conceptual frameworks and methodologies used by genomics science. In this vein, the editors of the Research Topic organised a cross-disciplinary roundtable on “genetic ancestry and the colonial legacies of race in genomics” (Schwartz-Marin et al.). It brought together geneticists and critical sociologists, across the global North and South, to debate the salience of race in genomics today, and sketch the contours of a more racially just genomics.

Other contributions to the Research Topic focused on the conduct of genomics studies. Gooden open consent framework for a South African genomics database stresses upon greater transparency in communicating the privacy risks of open access data, alongside deeper community engagement and a “higher-than usual benchmark” for informed consent. Going further in recognising that “social dynamics [perpetuate] colonial hierarchies”, Arango-Isaza et al. report on a new approach. In their recent genomic study with Mapuche communities in Chile, they shared the results with community members and incorporated their perspectives in the interpretation and final publication. Their contribution reflects on the challenges faced in knowledge sharing and addressing “economic and epistemological asymmetries”. Both these papers also contribute to developing guidelines for genomic research in global South contexts.

Crucially, large-scale genome sequencing initiatives demand significant economic and technical resources that are already constrained across the global South. They risk drawing away valuable funds from frontline health and development projects towards big, shiny technoscientific initiatives with unproven results. Additionally, they create technical and economic dependencies for developing countries (and communities) on multinational corporations, such as biotech, pharmaceutical or digital tech companies. These dependencies are criticised for infringing on communities’ data sovereignty (i.e., impeding data access and use by local researchers) and for reinforcing the shackles of intellectual property regimes that have contributed to scientific and health disparities in the first place.

In this regard, we organised a second cross-disciplinary roundtable to understand the bases of the “universal public good” in genomics (Jiwani et al.). While the global expansion of genomics is guided by an assumption of public good, we discussed the contradictions posed by the rampant commercialisation of genomic knowledge and data through the biotech juggernaut, and the structural asymmetries in access to genomic knowledge and innovations for the global majority.

This Research Topic sought to cultivate a more holistic engagement of genomics science with the ethical, social and political challenges it faces–and indeed its historical legacies–in the interest of forging a genomics that centers the question of justice. We invite readers from diverse perspectives to continue the conversation and to reach out to us with any feedback.

